# Clinical outcomes of two-stage revision total knee arthroplasty in infected cases with antibiotic-loaded cement spacers produced using a handmade silicone mold

**DOI:** 10.1186/s43019-021-00113-3

**Published:** 2021-08-28

**Authors:** Takashi Hoshino, Toshifumi Watanabe, Yusuke Nakagawa, Hiroki Katagiri, Nobutake Ozeki, Toshiyuki Ohara, Mikio Shioda, Yuji Kono, Ichiro Sekiya, Hideyuki Koga

**Affiliations:** 1 Department of Orthopaedic Surgery, Tokyo Medical and Dental University Hospital of Medicine, 1-5-45 Yushima, Bunkyo-ku, Tokyo, 113-8519 Japan; 2 Department of Joint Surgery and Sports Medicine, Graduate School, Tokyo Medical and Dental University, 1-5-45 Yushima, Bunkyo-ku, Tokyo, 113-8519 Japan; 3 Second Department of Orthopaedic Surgery, Dokkyo Medical University Saitama Medical Center, 2-1-50 Minami-Koshigaya, Koshigaya-shi, Saitama, 343-8555 Japan

**Keywords:** Periprosthetic joint infection, Total knee arthroplasty, Two-stage revision, Cement spacer, Hand-made silicon mold

## Abstract

**Purpose:**

This study assessed the clinical outcomes of periprosthetic joint infection patients who underwent two-stage revision total knee arthroplasty with antibiotic-loaded cement spacers fabricated using a handmade silicone mold.

**Materials and methods:**

This study included seven patients (average age 77 years, average follow-up time 54 months) who underwent surgery at our hospital between 2009 and 2013. Clinical outcomes including knee scores, function scores, knee range of motion, and walking ability at the final observation, period from the primary total knee arthroplasty to implant removal, period from implant removal to revision total knee arthroplasty, and follow-up period after revision total knee arthroplasty were investigated.

**Results:**

At the final follow-up, the average knee range of motion was 99°, with no significant differences at each stage; average knee and function scores were 84 and 77, respectively. With cement spacers, five patients were able to walk with a t-cane. No recurrence of infection was observed.

**Conclusions:**

The clinical outcomes of the current case series demonstrated good knee function with preserved walking ability, without any recurrence of periprosthetic joint infection. This study suggests that using a handmade silicone mold could be an effective option for periprosthetic joint infection after a total knee arthroplasty.

## Introduction

Periprosthetic joint infection (PJI) is a serious complication after total knee arthroplasty (TKA), and its incidence after primary TKA has been reported to be 1–2% [1, 2]. Previous studies have reported that PJI was one of the main reasons for the failure of primary TKA, with problems of reinfection and lowered patient activity levels [3]. Therefore, the appropriate prevention and treatment of PJIs are crucial.

Two-stage revision is considered the gold standard treatment for chronic PJI after TKA. A recent international consensus committee suggested that articulating spacers were better than static spacers during knee resection arthroplasty without major bone loss, lack of ligamentous integrity, or major soft-tissue defects [4]. Articulating spacers have some advantages such as maintaining joint space, maintaining mobility, preventing soft-tissue atrophy, and preserving walking ability [5–7].

A previous study has demonstrated that there was no difference in the success rates between several types of spacers [8]. However, a recent meta-analysis indicated that all-cement articulating spacers have a lower postoperative reinfection rate than prosthetic articulating spacers [9]. In addition, there are different ways of making cement molds, including commercially available molds and handmade molds [10–12]. However, the optimal choice of articulating spacer remains undetermined.

Two-stage revision TKA with antibiotic-loaded cement spacers using a handmade silicone mold was performed at our hospital; the method is easy and economical, and the cement implant size can be adjusted. The purpose of this study was to introduce our methods and to examine the midterm outcomes of PJI cases after primary TKA treated with antibiotic-loaded cement spacers produced using a handmade silicone mold. The hypotheses of this study was that antibiotic-loaded cement spacers produced using handmade silicone molds would be effective for PJI and preserve knee function.

## Material and methods

This study was approved by the Institutional Review Board, and written informed consent was obtained from all the patients. The patients gave their consent for publication of the data concerning the case. All surgeries were performed by four experienced orthopedic surgeons (TaM, TW, NS, ToM), and four surgeons evaluated the clinical outcomes.

### Patients

In the current study, patients who underwent surgery with antibiotic-loaded cement spacers produced using handmade silicone mold at the hospital between 2009 and 2013 and revision TKA later and who were followed up at the hospital were included. The exclusion criteria were as follows: (1) only irrigation and debridement cases and (2) cement implant was performed, but revision TKA was not performed. The knee scores, function scores, knee range of motion, and walking ability before the first-stage (first) surgery, before the second-stage (second) surgery, and at the final observation, along with the period from the primary TKA to implant removal, period from implant removal to revision TKA, and follow-up period after revision TKA were investigated.

### Treatment strategy

Patients who had undergone primary TKA at our hospital or other hospitals during the research period were diagnosed with PJI based on physical examination, local findings, laboratory data, synovial-fluid culture tests, and imaging tests. If a patient was taking antibiotics, the antibiotics were discontinued, and the synovial fluid was collected for bacteriological culture.

**Fig. 1:**
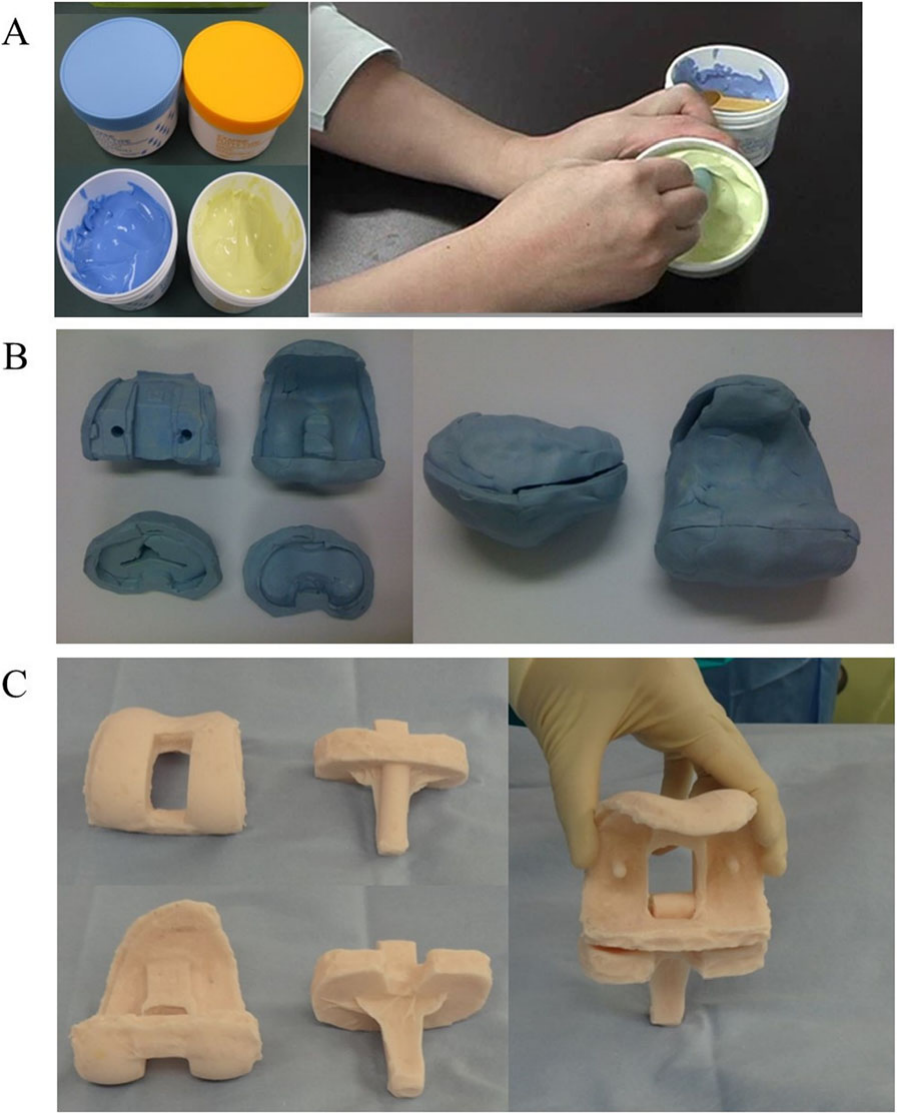
The process of manufacturing antibiotic-loaded articulating cement spacers. **A** Hydrophilic vinyl polysiloxane impression material was used. **B** A handmade silicone mold was made before surgery using trial components. C An antibiotic-loaded articulating cement spacer was made intraoperatively using the handmade silicone mold

Before the first surgery, handmade silicone molds of the same size as the primary TKA (Fig. 1A, B) were fabricated. A hydrophilic vinyl polysiloxane impression material (Exafine putty; GC Corporation, Tokyo, Japan), which is a popular impression material used in dental science, was used. Trial components were used to create handmade silicone molds.

**Fig. 2:**
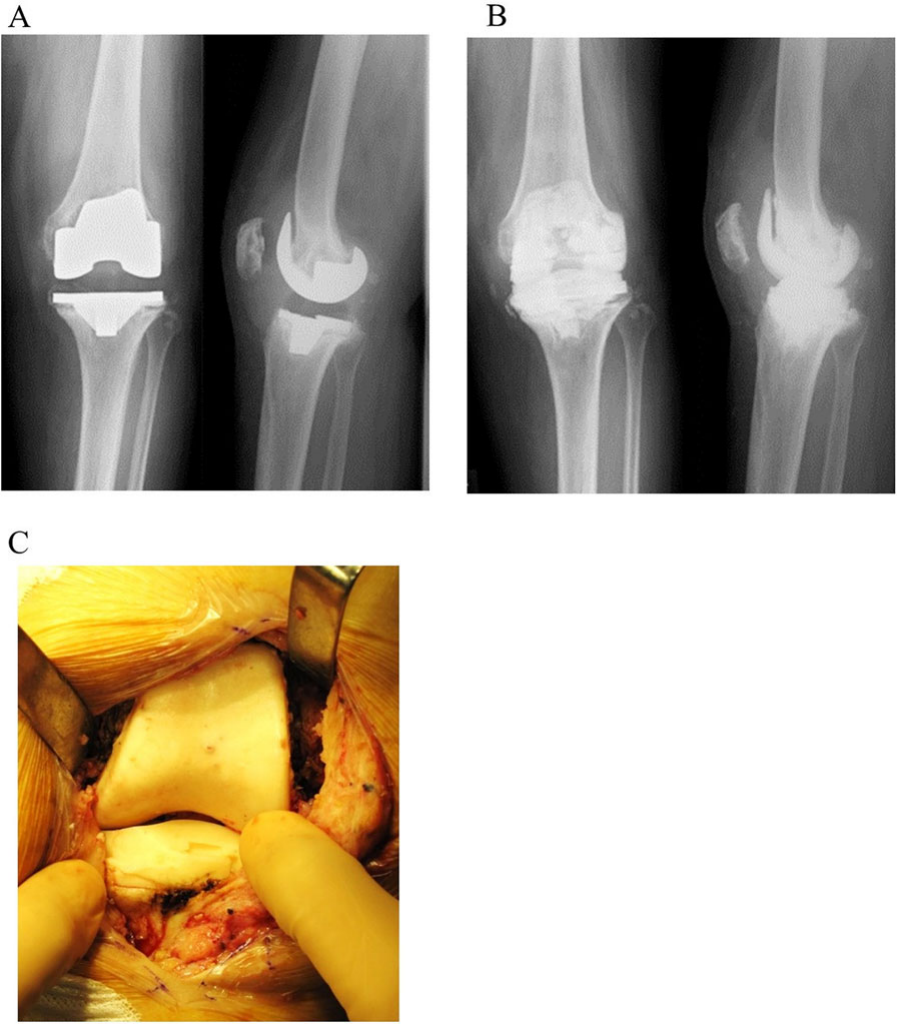
First-stage surgery: replacing antibiotic-loaded articulating cement spacers for periprosthetic joint infection. **A** Radiograph before the first surgery. Implant loosening was clearly observed. **B** Radiograph before the second surgery. Antibiotic-loaded articulating cement spacers were implanted. **C** Macroscopic image of articulating cement spacers

At the first surgery, infected synovial fluid and synovium were collected intraoperatively and submitted for bacteriological culture. After removing the primary implants, the infectious soft tissue including the synovium and cement were debrided. Thereafter, the whole infected knee was irrigated with 6 L or more of normal saline [13], and soaked with 1 L of 0.35% povidone-iodine for 3 min [14]. Finally, antibiotic-loaded cement spacers were implanted (Fig. 2A-C). The antibiotic-loaded cement spacers were fabricated using a handmade silicone mold during surgery (Fig. 1C). Cemex bone cement (Tecres, Verona, Italy) was used because of its low maximum polymerization temperature [15]. Bone cement was mixed with 2 g vancomycin and 180 mg tobramycin per 40 g of each package [16].

Postoperatively, full weightbearing, quadriceps muscle setting, and range of motion exercises were started the day after surgery, and gait exercises were encouraged 3–4 days postoperatively [17]. Patients received intravenously administered antibiotic therapy for about a week, until laboratory data were almost normalized (white blood cell count, normal; C-reactive protein level, < 1.0 mg/dL; erythrocyte sedimentation rate, reduced to normal range). Subsequently, patients were given orally administered antibiotics continuously for approximately 3 months. The orally administered antibiotics were selected based on culture results. The wound status, radiographic findings, and laboratory data were observed regularly.

The second revision surgery was scheduled when the infection had beens controlled, as confirmed by normalized local findings, laboratory data, and a negative bacteriological culture of the joint fluid after at least 2 weeks of antibiotic discontinuation. A negative culture, at least twice, was mandatory. In the case of rheumatoid arthritis (RA), if the laboratory data were not negative, normalized local findings and negative cultures were confirmed at least twice.

**Fig. 3:**
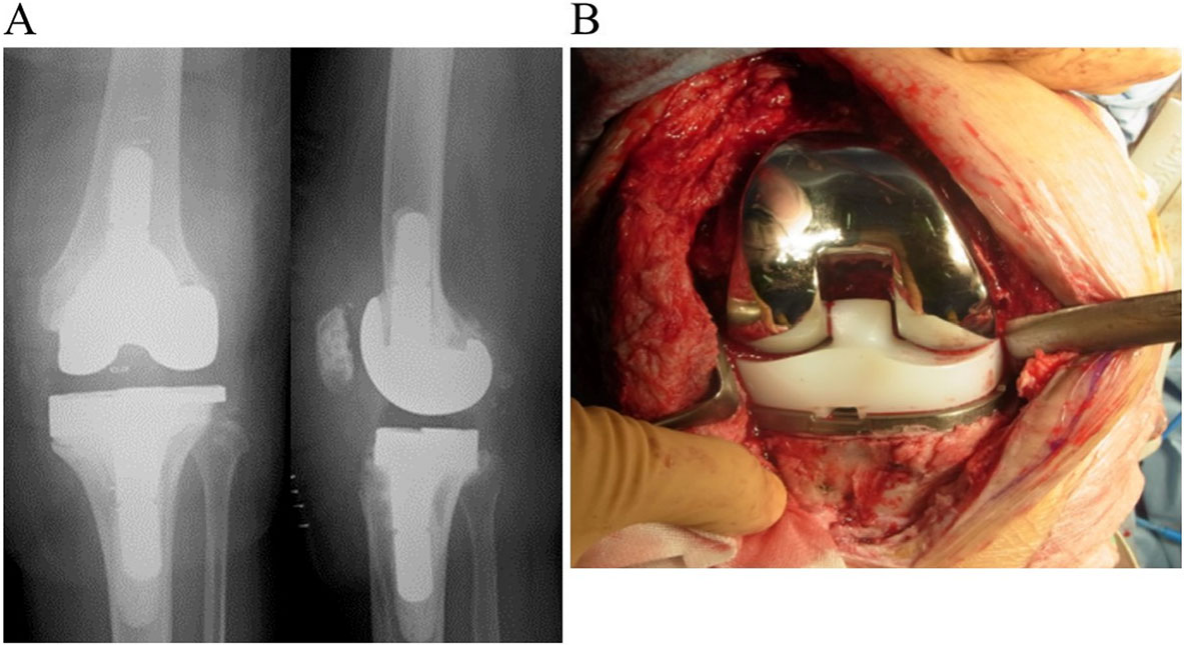
Second-stage surgery: revision total knee arthroplasty (TKA). **A** Radiograph at final follow-up. Revision TKA was completed without implant loosening. **B** Macroscopic image of TKA prosthesis

During the second surgery, the synovium was collected intraoperatively; if there were fewer than five leukocytes in a high-power field by intraoperative cell count, revision surgery was performed as scheduled (Fig. 3 A-B). Contrastingly, an illustration of five or more leukocytes indicated the presence of infection, and the antibiotic-loaded cement spacer was placed in position again. However, there were no cases of cement spacer reimplantation in the current study. NexGen® LCCK system (Zimmer, Warsaw, IN, USA) was used for the second revision surgery. Orally administered antibiotics are not usually administrated after revision TKA .

### Statistics

Statistical analyses were performed using IBM SPSS (Version 26.0, IBM Corp, Armonk, NY, USA). Knee range of motion and knee society scores before the first and second surgeries, and at the final observation were compared using the Friedman test followed by the Bonferroni correction for multiple tests. Post hoc power analysis for nonparametric tests was performed using G-power 3.1 calculation software (Kiel University, Kiel, Germany) as described previously [18], and it revealed that, with an alpha of 0.05, a power of 0.68 was achieved for knee score. A *P* value < 0.05 was considered significant.

## Results

**Fig. 4:**
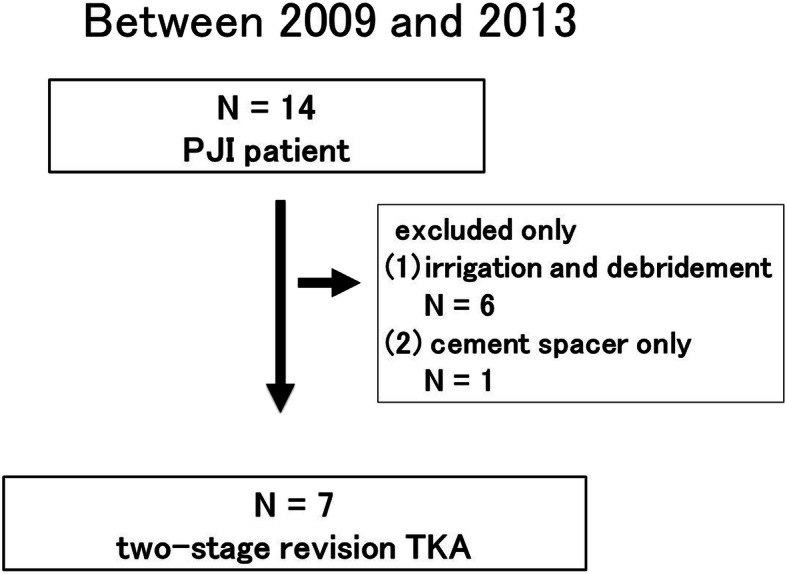
Flowchart showing the selection of patients for analyses

Between 2009 and 2013, 14 patients with PJI were treated at this hospital. Six patients underwent only irrigation and debridement, and one patient did not undergo revision TKA since he did not want to undergo this owing to his unstable general condition and because he was able to walk with a cement implant. After applying all the exclusion criteria, seven patients were finally included in the study (Fig. 4).

The patients’ demographic and clinical data are summarized in Table 1. The patients included four women and three men. The cultured organisms included methicillin-resistant *S. aureus* (MRSA), *E. faecalis*, α-streptococcus, β-streptococcus, *S. capitis*, *S. bovis*, and various unknown bacteria. The RA patient’s C-reactive protein level and erythrocyte sedimentation rate were improved but did not return to negative. The revision TKA was performed after confirming normalized local findings and negative culture twice.

**Table 1:** Patient’s demographic data

Case	Age at the 1^st^ surgery	Gender	Diagnosis	Organism	Past medical history
1	81	Female	OA	E.faecalis	HT, DL
2	70	Female	OA	α-streptococcus	None
3	85	Female	OA	Unknown	Cholecystitis
4	75	Female	OA	S.capitis	DL
5	80	Male	RA	S.bovis	Stomach cancer, HT
6	82	Male	OA	β-streptococcus	OMI
7	76	Female	OA	MRSA	DM, HT

Abbreviations: *OA* Osteoarthritis, *RA* Rheumatoid arthritis, *E.faecalis* Enterococcus faecalis, *MRSA* Methicillin‐resistant Staphylococcus aureus, *S.captitis* Staphylococcus capitis, *S.bovis* Streptococcus bovis, *HT* Hypertension, *DL* Dyslipidemia, *OMI* Old myocardial infarction, *DM* Diabetes

Patients’ clinical data are presented in Table 2. The average time from primary TKA to cement spacer implantation was 28 ± 16 months (range, 10–53 months). The average time from cement spacer to revision TKA was 6 ± 3 months (range, 3–12 months). During follow-up, one of the seven patients died from other non-related diseases. The average follow-up time after revision TKA was 54 ± 28 months (range, 11–90 months). There were no cases of cement spacer reimplantation in the current study, and all revision cases had no recurrence of infection at the final follow-up.

**Table 2:** Patient’s clinical data

									Knee society score					
	Interval (month)		F/U (month)	Range of motion			Walking ability		Knee score			Function score		
Case	Pre -1st	1st-2nd	Final	Pre-1st	Pre-2nd	Final	1st-2nd	post 2nd	Pre-1st	Pre-2nd	Final	Pre-1st	Pre-2nd	Final
1	23	7	77	-8 - 50	-10 - 100	0 - 100	T-cane	Independent	28	68	92	30	55	90
2	23	5	48	10 - 85	0 - 100	0 - 90	T-cane	Independent	32	70	73	30	55	90
3	53	12	11	-2 - 115	0 - 120	3 - 135	T-cane	T-cane	43	79	95	15	45	85
4	13	5	90	-2 - 85	-2 - 70	10 - 70	Walker	Walker	37	59	67	5	35	50
5	46	3	26	0 - 120	15 - 100	12 - 125	Double-cane	T-cane	49	70	87	15	25	75
6	28	4	64	0 - 90	0 - 80	0 - 100	T-cane	T-cane	43	71	88	20	30	75
7	10	7	60	5 - 90	10 - 80	0 - 100	T-cane	T-cane	43	76	88	30	55	75
Mean ± SD	28 ± 16	6 ± 3	54 ± 28	90 ± 22	91 ± 19	99 ± 22	-	-	39 ±7	70 ± 6	84^a^ ± 10	21 ± 10	43 ± 13	77^b^ ± 14
Range	10-53	3-12	11-90	58-120	70-120	60-132	‘-	‘-	28-49	59-79	67-92	5-30	25-55	50-90

Abbreviations: *Pre* Pre-operation, *1*^*st*^ First stage surgery, *2*^*nd*^ Second stage surgery, *Final* Final observation, *F/U* Follow-up

^a^*P* < 0.01, vs Pre 1^st^ surgery

^b^*P* < 0.01, vs Pre 1^st^ surgery

The average knee range of motion was 90° ± 22° before the first surgery, 91° ± 19° before the second surgery, and 99° ± 22° at the final follow-up. There was no significant difference in the range of motion between the surgeries.

Regarding the knee society scores, the average knee score was 84 ± 10, and the function score was 77 ± 14 at the final follow-up. Both scores significantly improved between surgeries.

The patients who received cement spacers before the second surgery were able to walk in five cases with a t-cane, in one case with double t-cane, and in one case with a walker. The walking ability was maintained after the first surgery.

## Discussion

The most important finding of the current study was that the range of motion was preserved and most of the patients could walk with a t-cane during cement spacer implantation. Knee function was maintained between the first and second surgeries. There was no recurrence of infection during the observation period.

In the current study, articulating all-cement spacers were used for patients with PJI. Articulating spacers have been commonly used for PJI and several types have been reported [16]. Further, the all-cement spacer is an articulating spacer. Several industrial preformed polymethylmethacrylate (PMMA) spacers, such as the Spacer-K® (Teres S.P.A, Verona, Italy) or the Inter Space Knee® (Exactech Inc., Gainesville, FL, USA) are available. These commercially manufactured antibiotic-containing spacers are designed as ultra-congruent condylar knee prostheses. Castellis et al. [19] reported that articulating spacers for infected TKA improved patient quality of life between stages and reduced social costs. Although preformed spacers may reduce the operating time, they have problems such as limited size variations and the inability to fit all patients’ knees. In addition, they contain limited dose of antibiotics and are more expensive than other spacers. Another option for the articulating spacer is the use of a handmade spacer made using intraoperative molds, such as commercial molds, bone cement molds, and silicone molds. For instance, it is easy to make a cement spacer from commercial molds, such as Stage One® (Biomet, Warsaw, IN, USA) [12], however, these molds are expensive and have limited size variations. These spacers do not have a post-cam design or stem. However, Stephen et al. [20] modified the technique and attached a rod covered with antibiotic-impregnated cement to the spacer. Regarding the intraoperative mold, Shen et al. [21] reported that they made a custom mold intraoperatively with bone cement using trial components. The advantage of this method is that a mold of the same size as the original prosthesis can be made; however, sterile paraffin oil is needed to prevent adherence of the cement to the mold. Regarding the handmade silicone mold, Durbhakula et al. [10] reported on the vacuum-injected silicone mold. Su et al. [22] reported a technique similar to ours that revealed good clinical outcomes without recurrence of infection.

Compared with other studies using the silicone mold, a hydrophilic vinyl polysiloxane impression material that is popular in dental science was used in this study; this putty-type material allows for easy fabrication of the mold and is cheaper than the commercial mold. Su et al. also used a putty-type silicone impression material. [22]. Their features are similar to those of this method, although there is a difference in the bone cement. The Cemex bone cement used in this study had a low maximum polymerization temperature and, therefore, a low risk of antibiotic deactivation [15].

A combination of two antibiotics in acrylic bone cement spacers for PJI is the gold standard treatment. A previous study has demonstrated that the combination of vancomycin and tobramycin in bone cement increased the elution of both antibiotics from bone cement [23]. Another study reported that antibiotic loading in higher doses in acrylic bone cement did not necessarily lead to enhanced antibiotic elution [24]. In this study, we mixed 2 g vancomycin and 180 mg tobramycin into 40 g of one bone cement package. There were no cases of infection recurrence or breakage of cement spacers indicating that the choice of antibiotic ratio was effective.

Regarding knee function, the current study illustrated that walking ability and knee range of motion were maintained before and after the second surgery. A previous review indicated that the articulating spacer group had a significantly higher range of motion than the static spacer group [25]. Durbhakula et al. demonstrated that minimal soft-tissue contracture and minimal bone loss were encountered during articulation of cement spacers [10]. Castelli et al. reported that the mean ROM was 77° (range, 10–100°) and 77% of patients used only one crutch, and approximately 40% of patients with articulating spacer were capable of walking without crutches [19]. The results of this study are comparable to those of previous related studies. Furthermore, we allowed patients to walk fully weightbearing with a cement spacer that might have contributed to their ability to walk. Considering the average age of 82 years at follow-up, the knee function of the patients was well maintained throughout the treatment.

Regarding reinfection after revision TKA, some previous studies have reported a high success rate of two-stage revision TKA, although reinfection cases were observed more than 7 years after revision TKA. [26–28]. Mortazavi et al. reported that culture-negative or methicillin-resistant PJI increased the risk of failure [29]. In this study, there was one case of MRSA and one culture-negative case. During the follow-up period, although there were no cases of reinfection, careful long-term observation of such cases is essential.

This study had several limitations. First, the sample size was small. The current study was not sufficiently powered for clinical outcomes and, thus, was likely underpowered in detecting them, although there was a tendency for clinical results to improve. Second, the follow-up period ranged from short to medium. A longer follow-up study with more patients is necessary to verify the results of this study. Third, as revision surgeries were performed by four surgeons, surgical results and assessments might be heterogeneous. Finally, the current study was a retrospective case series, and this hospital was a tertiary referral hospital that could have introduced selection and recall bias. Four patients in this study underwent primary TKA in other hospitals.

The strength of this method is that it is an easier and more economical cement spacer than the commercially available molds. Furthermore, the cement spacers used in this study depicted clinical outcomes comparable to those of other spacers. Therefore, the clinical relevance of this study is that this method could be a treatment option for PJI cases.

## Conclusions

Clinical outcomes of PJI cases after two-stage revision TKA with antibiotic-loaded cement spacers produced using a handmade silicone mold revealed good knee function with preserved walking ability without recurrence of PJI. This study suggests that using a handmade silicone mold could be an effective option for PJI after TKA.
